# Pathogenic mechanisms and potential applications of extracellular vesicles from periodontal pathogens in periodontitis

**DOI:** 10.3389/fimmu.2024.1513983

**Published:** 2024-12-20

**Authors:** Ruiqing Zhang, Guoliang Li, Yingtao Wu, Xiaoxuan Wang, Qingxian Luan

**Affiliations:** ^1^ Department of Periodontology, Qingdao Stomatological Hospital Affiliated to Qingdao University, Qingdao, Shandong, China; ^2^ Department of Periodontology, Peking University School and Hospital of Stomatology & National Center for Stomatology & National Clinical Research Center for Oral Diseases & National Engineering Research Center of Oral Biomaterials and Digital Medical Devices & Beijing Key Laboratory of Digital Stomatology & NHC Key Laboratory of Digital Stomatology & NMPA Key Laboratory for Dental Materials, Beijing, China; ^3^ Department of Radiation Oncology, The Affiliated Hospital of Qingdao University, Qingdao, Shandong, China

**Keywords:** periodontal pathogen, periodontitis, bacterial extracellular vesicles, outer membrane vesicles, periodontitis pathogenesis, BEVs application

## Abstract

Periodontitis is a multifactorial disease characterized by chronic destruction of the periodontal supporting tissues and is closely associated with the dysbiosis of the plaque biofilm. It is the leading cause of tooth loss in adults. Bacterial extracellular vesicles (BEVs) are released from bacteria, which range in size from 20 to 400 nm. These vesicles contain various components derived from their parent bacteria, including nucleic acids, proteins, lipids, and other molecules, which facilitate functions such as molecular transfer, metabolic regulation, bacterial interactions, biofilm formation, and immune modulation. BEVs participated in the pathophysiological process of periodontitis. Recently emerging evidence also showed that the contents of EVs in saliva and gingival crevicular fluid (miRNAs, mRNAs, and proteins) could be used as potential biomarkers for periodontitis. While most current research focuses on human-derived components, much less is known about BEVs. Therefore, this review introduces the formation mechanisms and components of BEVs related to periodontitis. Then, this review summarizes the current information about the mechanism, the diagnostic and theraputic value of periodontal pathogen-derived extracellular vesicles in the development of periodontitis. Furthermore, the future challenges of exploring the role of BEVs in periodontitis are also discussed.

## 1 Introduction

Periodontitis is a common chronic inflammatory condition, primarily characterized by the progressive destruction of the periodontal ligament and alveolar bone. It has a high prevalence, affecting more than 60% of adults worldwide, while the prevalence of severe periodontitis is over 20% (1, 2). This condition imposes a significant economic and health burden on patients, severely impacting their quality of life (3, 4). Periodontitis is recognized as a multifactorial disease, closely linked to dysbiosis within the plaque biofilm. The complex crosstalk between multiple pathogenic microorganisms and the host immune system plays a crucial role in the pathogenesis of periodontitis (5–7). However, the exact mechanisms has yet to be fully elucidated. In the absence of timely intervention and treatment, periodontitis has the potential to induce tooth mobility and even tooth loss. By now, periodontits has become the leading cause of tooth loss in adults and may also trigger systemic inflammatory responses (2, 8). Early-stage periodontitis often remains undiagnosed due to subtle symptoms and limitations in radiographic imaging, and once the disease progresses, periodontal tissue regeneration treatments may yield less-than-ideal outcomes. Thus, addressing the potential pathogenic mechanisms, diagnostic methods, and prevention and treatment strategies for periodontitis remains an urgent challenge in periodontal care.

Extracellular vesicles (EVs) are nanoscale particles enveloped by a lipid bilayer, released by both host and microbial cells, including bacteria and fungi (9). As the concept of the human microbiome in relation to health and disease becomes mature gradually, there is an increasing recognition of microbe-derived EVs, specifically bacterial EVs (BEVs) and their function in facilitating communication between microbes and their hosts (10). BEVs contain various components derived from their parent bacteria, including nucleic acids, proteins, lipids, and other molecules, which facilitate functions such as molecular transfer, metabolic regulation, bacterial interactions, biofilm formation, and immune modulation (11). Pathogenic BEVs play a pivotal role in enhancing pathogenicity due to their small size, structural stability, inclusion of multiple virulence factors, and ability to evade immune detection and facilitate distant dissemination (12, 13). They are considered key novel mediators of bacterial interaction, either between bacteria themselves or with the host.

This review provides an overview of biogenesis and classification of BEVs related to periodontitis and their pathogenic mechanisms in periodontal diseases. Moreover, this review also summarizes the diagnostic and theraputic value in the development of periodontitis. Furthermore, the future challenges of exploring the role of BEVs in periodontitis were also discussed. The review may offer new insights into the role of periodontal pathogen BEVs in disease progression and the development of novel strategies for periodontal diagnosis and therapy.

## 2 Biogenesis and classification of BEVs

Both Gram-negative and Gram-positive bacteria produce extracellular vesicles (BEVs), which range in size from 20 to 400 nm (14). The composition and mode of BEVs production vary among different bacteria. Gram-positive bacteria typically produce cytoplasmic membrane vesicles (CMVs), while Gram-negative bacteria secrete outer membrane vesicles (OMVs) (15).

### 2.1 BEVs produced by Gram-negative bacteria

**Figure 1:**
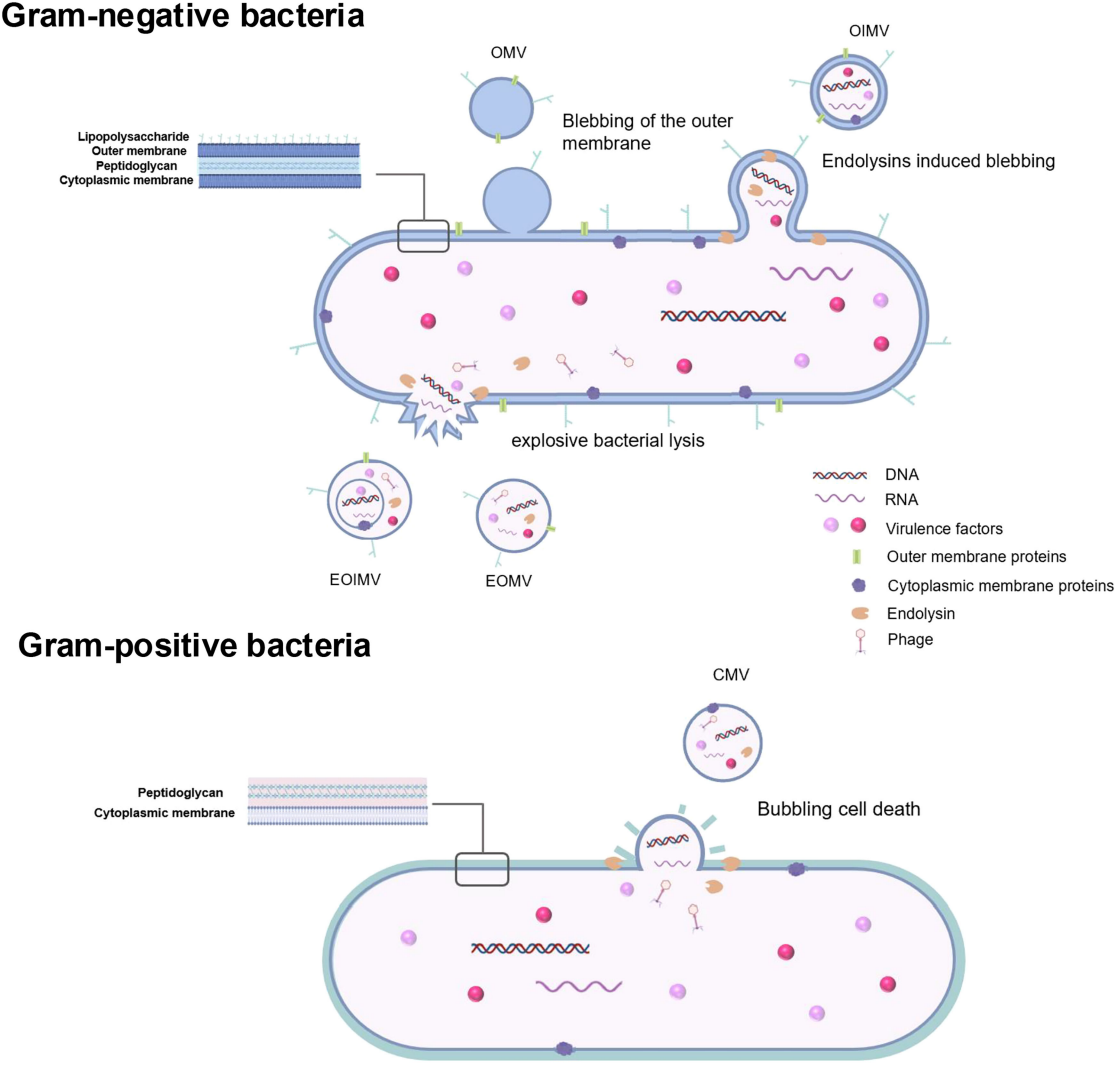
Extracellular vesicles produced by Gram-negative bacteria and Gram-positive bacteria. As for Gram-negative bacteria, blebbing of the outer membranes and explosive bacterial lysis are two main mechanisms in BEVs formation. Typical OMVs generated by Gram-negative periodontopathogens are produced by blebbing of the outer membranes without carrying cytoplasmic components. OIMVs are formed by autolysin and contain cytoplasmic components. EOMVs and EOIMVs are produced by phage-derived endolysin and contain cytoplasmic contents from cells exposive. Gram-positive bacteria which lack an outer membrane generate extracellular vesicles called CMVs mainly by explosive cell lysis. *OMVs outer membrane vesicles, OIMVs outer-inner membrane vesicles, EOMVs* explosive outer *membrane vesicles, explosive outer-inner membrane vesicles, CMVs cytoplasmic membrane vesicles.*.

Although a considerable body of research has been carried on the formation of BEVs, the underlying mechanisms remain incompletely understood. Key periodontal pathogens are mostly Gram-negative obligate and facultative anaerobic bacteria, including *Porphyromonas gingivalis (P. gingivalis), Fusobacterium nucleatum (F. nucleatum), Aggregatibacter actinomycetemcomitans (A. actinomycetemcomitans), Tannerella forsythia (T. forsythia), and Treponema denticola (T. denticola)* (16). The cell walls of Gram-negative bacteria are composed of lipopolysaccharides (LPS) and a thin layer of peptidoglycan, characterized by an outer membrane and an inner membrane separated by the periplasm. The production of Gram-negative bacterial membrane vesicles (MVs) involves mechanisms such as non-lytic vesicle formation (Type B) and explosive cell lysis with subsequent membrane fragment fusion into vesicular structures (Type E) (17). Bacterial outer membrane vesicles (OMVs) represent the primary form of BEVs released by periodontal pathogens. OMVs formation occurs when the expansion of the outer membrane outpaces the peptidoglycan layer, often due to the loss or repositioning of covalent bonds between these two structures (18) (**Figure 1**). Additionally, peptidoglycan fragments or misfolded proteins may exert expansive pressure on the outer membrane, leading to OMVs formation (19, 20). Specific phospholipids, LPS, and other molecules that accumulate in the bacterial outer membrane can also induce OMVs production by altering membrane curvature (21). For example, OMVs formation in *P. gingivalis* may be facilitated by the upregulation of certain inner or outer leaflet lipids, linked to the selective incorporation of anionic lipopolysaccharides (A-LPS) and the C-terminal domain (CTD) family of proteins on the bacterial surface (22). Another proposed mechanism involves the VacJ/Yrb ABC phospholipid transporter system, which may regulate OMVs formation (23). OMVs lack DNA and RNA but are enriched in periplasmic proteins and lipids. Conversely, outer-inner MVs contain cytoplasmic components, likely due to the weakening of the bacterial peptidoglycan layer by endolysins (12, 21) (**Figure 1**). The expression of phage-related endolysins can degrade the peptidoglycan layer, leading to explosive bacterial lysis and the formation of Type E MVs, which include explosive OMVs (EOMVs) and explosive outer-inner MVs (EOIMVs), both containing cytoplasmic contents (24) (**Figure 1**).

### 2.2 BEVs Produced by Gram- positive bacteria

Among periodontal pathogens, Gram-positive bacteria are relatively rare. However, recent oral microbiome studies have highlighted *Filifactor alocis (F. alocis)*, a Gram-positive, obligate anaerobe, as an important periodontal pathogen (25). *F. alocis* has been frequently detected in chronic periodontitis, aggressive periodontitis and peri-implantitis (21, 26). Gram-positive bacteria, which possess a thick peptidoglycan cell wall, typically release EVs through explosive cell lysis, producing cytoplasmic MVs, a process referred to as “bubbling cell death.” (12, 27) (**Figure 1**).

## 3 Composition of periodontal pathogen-derived EVs

The composition of BEVs is influenced by the biogenesis mechanism, bacterial species, growth stage, and environmental conditions. Their cargo plays a critical role in disease progression, biofilm modulation, and immune evasion. However, the precise mechanisms governing cargo selection during BEVs release remain elusive. Periodontal pathogen-derived EVs contain proteins, lipids, nucleic acids and other biomolecules, and carry numerous virulence factors such as toxins, LPS, adhesins, and proteolytic enzymes, which contribute to periodontal diseases.

### 3.1 Proteins

Proteins found in BEVs originate from the outer membrane, periplasm, and cytoplasm of the parent bacteria (18). Proteomic analyses have revealed a diverse array of proteins in BEVs, including structural proteins, porins, and transporters involved in various biological processes (28–30). Under specific conditions, certain proteins may be preferentially loaded into EVs. Gingipains, lysine-specific (Kgp) and arginine-specific (RgpA) proteases, are major virulence factors in *P. gingivalis* OM and OMVs (31, 32). The absence of RgpA reduces OMV secretion (33). Studies comparing *P. gingivalis* OMVs and outer membrane protein cargo have shown that CTD proteins derived from gingipains are concentrated on OMVs and lipoproteins involved in iron acquisition are also selectively sorted into OMVs (34). Under conditions of hemin excess, some moonlighting cytoplasmic proteins, with adhesive potential, are preferentially loaded onto OMVs, promoting *P. gingivalis* proliferation and co-aggregation with other bacteria in specific environments (35). Peptidylarginine deiminase (PPAD), detected in *P. gingivalis* OMVs (36), has been shown to be associated with OMV biogenesis through citrullination activity (37). PPAD also facilitates immune evasion and has been implicated in autoimmune diseases, such as rheumatoid arthritis (38).

OMVs produced by *A. actinomycetemcomitans* are rich in leukotoxin, which selectively kills host immune cells (39, 40). LtxA can be selectively sorted into large OMVs(>300 nm) due to surface-associated DNA driving (41). Additionally, *A. actinomycetemcomitans* OMVs can deliver cytolethal distending toxin (CDT) to HeLa cells and human gingival fibroblasts (HGFs), causing the characteristic cytolethal distending effect (42). CDT, a genotoxin, induces DNA damage in mammalian cells, leading to G2 cell cycle arrest, progressive cell enlargement, and/or apoptosis (43, 44). CDT toxicity has been linked to GSK-3-dependent cell cycle arrest in gingival keratinocytes (45).

OMVs derived from *T. denticola* contain adhesins and serine proteases necessary for adhering to and degrading host cells and mammalian matrix proteins (46). OMVs from *T. forsythia* harbor several virulence factors, including leucine-rich-repeat family virulence factor BspA, a Toll-like receptors 2 (TLR2) agonist, as well as non-TLR2 agonist virulence factors such as sialidase and GroEL (47).

### 3.2 Lipids and lipopolysaccharides

Lipids are important structural components of BEVs, but their specific composition remains understudied. It has been shown that *P. gingivalis* can synthesize sphingolipids, which are delivered via OMVs and suppress host immune responses (48, 49). Lipid rafts play a crucial role in OMV-mediated endocytosis by host cells, as seen in both *P. gingivalis* and *A. actinomycetemcomitans* OMVs (42, 50).

LPS is the most abundant surface antigen in Gram-negative bacteria and also a critical structural and toxic component of BEVs (51). LPS consists of lipid A, a core oligosaccharide, and an O-antigen polysaccharide chain (52). *P. gingivalis* expresses two types of LPS: neutral O-LPS and anionic A-LPS, the latter of which is involved in OMV formation (22, 53). In addition, *T. f*orsythia and *T. denticola* OMVs express low-molecular-weight lipooligosaccharides (54, 55).

### 3.3 Genetic material

BEVs carry genetic material, including DNA, mRNA, sRNA, and other non-coding RNAs from the parent bacteria, which can mediate horizontal gene transfer (HGT) between species (56). HGT is a crucial driver of gene and genome evolution (57). DNA and RNA have been detected in OMVs from *P. gingivalis*, *T. denticola*, and *T. f*orsythia, meanwhile, they can activate TLR7, TLR8, and TLR9 receptors (54). Extracellular DNA (eDNA) has also been identified on OMV surfaces, forming an eDNA/OMV network that may aid in nutrient capture for pathogens residing on the surface of polymicrobial biofilms (54).

*A. actinomycetemcomitans*-derived OMVs contain extracellular RNA (exRNA), which is protected from enzymatic degradation in body fluids by encapsulation within the EVs (58). exRNA is transferred into host cells via OMVs and may be integrated into the host RNA-induced silencing complex, regulating host target transcripts. *A. actinomycetemcomitans* OMVs and exRNA influence not only local immune responses but may also cross the blood-brain barrier (59). Furthermore, *A. actinomycetemcomitans, T. denticola*, and *P. gingivalis* secrete small RNAs of microRNA size (miRNA-size, small RNAs or msRNAs) via OMVs, which are stably transferred to host cells, modulating immune responses and apoptosis (60, 61).

## 4 Roles of periodontal pathogen-derived BEVs in the progression of periodontitis

As BEVs serve as media of communication between bacteria and host cells, they can mediate the interaction between bacteria to affect plaque biofilm formation as well as interact with cell receptors or enter cells to exert pathogenic effects. The specific molecular mechanism of BEVs internalization by host cells remains to be further elucidated. Currently, there are several internalization pathways for EVs, including endocytosis, internalization through lipid rafts, membrane fusion, and receptor-mediated signal transduction (62). Endocytosis is the most common internalization pathway for BEVs internalization (63). Moreover, BEVs can also communicate with host cells through signal transduction mediated by toll-like receptors (TLRs) such as TLR2 and TLR4 (15). The internalization of BEVs triggers a series of responses in host cells, including immunomodulation and periodontal tissue destruction.

### 4.1 Regulation of plaque biofilm

**Figure 2:**
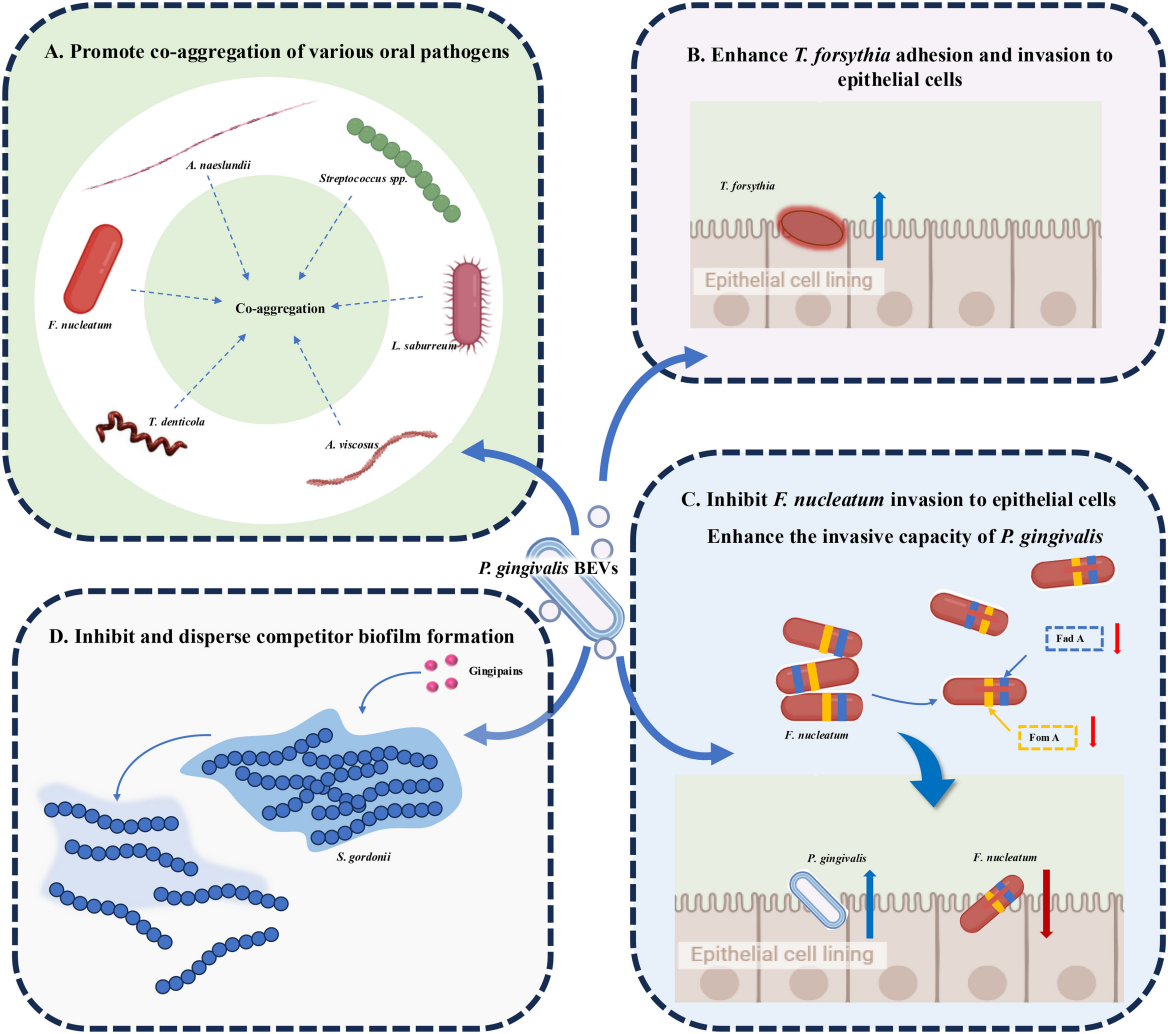
Roles of Periodontopathogen-Derived BEVs in Plaque Biofilm Regulation. **(A)** *P. gingivalis* OMVs aggregate *Streptococcus* spp.*, F nucleatum, A nucleatum, A viscosus, T. denticola* and *L. saburreum*. **(B)** *P. gingivalis* OMVs enhance the ability of *T. forsythia* to adhere to and invade epithelial cells. **(C)** *P. gingivalis* OMVs reduce FadA and FomA on the surface of *F nucleatum*, inhibiting its invasion of oral epithelial cells and its auto-aggregation. During co-infection, *F nucleatum* paradoxically enhances the invasive capacity of *P. gingivalis*. **(D)** *P. gingivalis* OMVs inhibit and disperse *S. gordonii* biofilm formation in a gingipain-dependent manner.

Plaque biofilm formation plays a central role in the onset and progression of periodontitis (64), and bacterial interactions are critical to the formation of subgingival biofilm. Periodontal pathogens can communicate with one another or with other bacteria via BEVs, influencing biofilm formation, bacterial survival, and enhancing their invasive capabilities (65) (**Figure 2**). A series of studies have shown that OMVs from *P. gingivalis*, enriched with gingipains or adhesins, significantly promote co-aggregation of various oral pathogens, such as *Streptococcus* spp., *F. nucleatum*, *Actinomyces naeslundii* (*A.naeslundii*), and *Actinomyces viscosus (A. viscosus)* (66). Additionally, *P. gingivalis* OMVs mediate co-aggregation with *T. denticola* and *Lachnoanaerobaculum saburreum* (*L.saburreum*), and enhance the motility of non-motile bacteria, aiding the formation of a multispecies plaque biofilm (67). Moreover, *P. gingivalis* OMVs enhance *T. forsythia* adhesion to and invasion of epithelial cells, contributing to its virulence (68). Zhang (69) et al. reported that proteases in *P. gingivalis* OMVs reduce the expression of adhesion-associated proteins, such as FadA and FomA, on the surface of *F. nucleatum*, inhibiting its invasion of oral epithelial cells and its auto-aggregation. However, it has no impact on the morphology or proliferation of *F. nucleatum*. During co-infection with *F. nucleatum* and *P. gingivalis*, *F. nucleatum* paradoxically enhances the invasive capacity of *P. gingivalis*. By preventing *F. nucleatum* degradation within cells and maintaining its bioactivity, *P. gingivalis* promotes deeper infection. Furthermore, periodontal pathogen-derived BEVs have the function of inhibiting and dispersing competitor biofilms. For example, *P. gingivalis* OMVs appeared to have a negative impact on biofilm formation and the maintenance of *Streptococcus gordonii (S. gordonii)* in a gingipain-dependent manner, creating a more favorable environment for its own survival (70).

### 4.2 Immunomodulation

**Figure 3:**
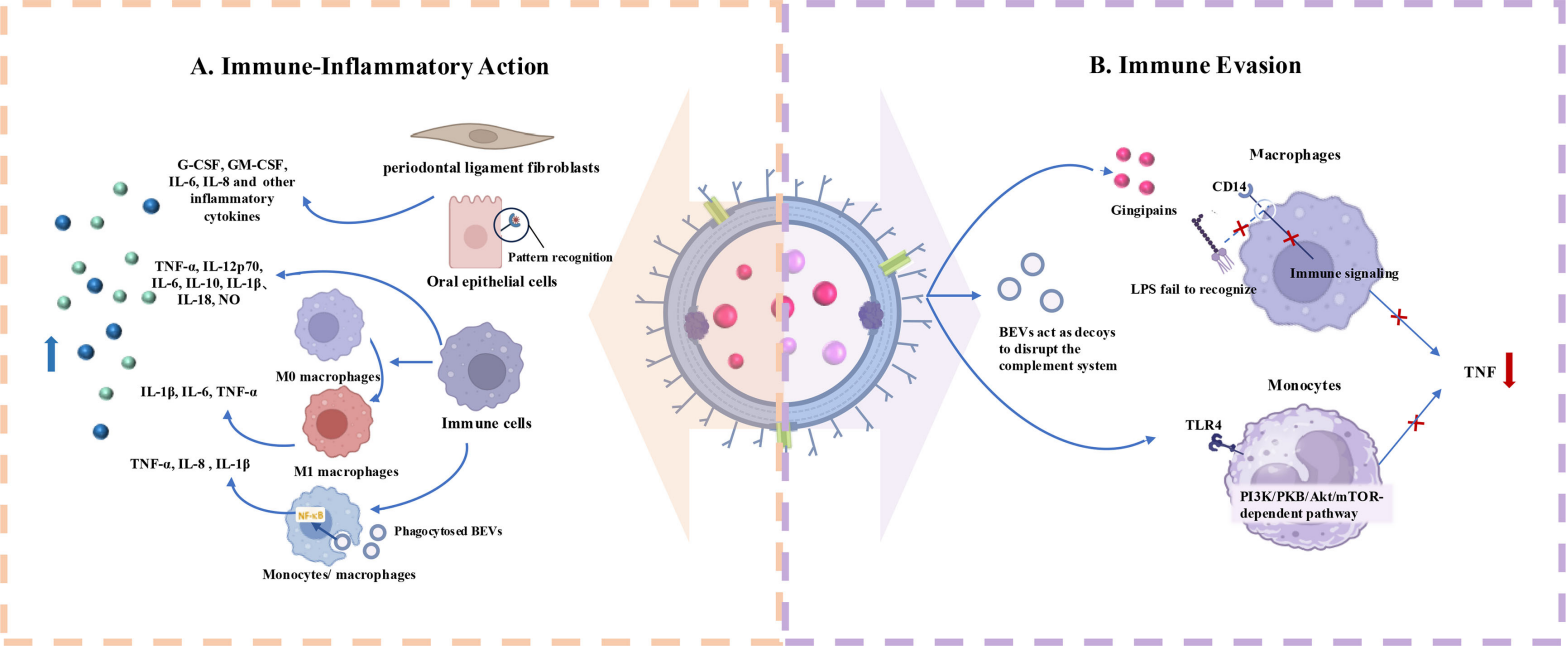
Roles of Periodontopathogen-Derived BEVs in Immunomodulation. **(A)** Periodontopathogen-Derived BEVs induce immune-inflammatory responses in host cells including immune and non-immune cells. **(B)** BEVs help periodontal pathogens escape from host immune system by acting on immune cell membrane receptors and serving as decoys to consume complement components.

BEVs from periodontal pathogens have significant immunomodulatory effects on host cells including both triggering the activation of the immune system and contributing to immune evasion (**Figure 3**).

#### 4.2.1 Induction of immune-inflammatory responses

Periodontitis is an immune-inflammatory reactive disease initiated by plaque biofilm (71). EVs produced by various periodontal pathogens play a pivotal role in inducing host immune-inflammatory responses similar to their parent bacteria, thereby contributing to the pathogenesis of periodontitis (72). Host cells that internalize BEVs and trigger immune responses include both immune and non-immune cells.

Cecil (54) et al. found that EVs produced by periodontal pathogens can activate TLRs and nucleotide-binding oligomerization domain (NOD) pattern recognition receptors (PRRs) in gingival epithelial cells, potentially triggering significant inflammatory responses via multiple signaling pathways. Another study demonstrated that *P. gingivalis* OMVs activate Erk1/2, JNK, MAPK, STING, and NF-κB signaling pathways, leading to enhanced expression of interleukin (IL)-6 and IL-8 in human gingival epithelial cells (73). *F. alocis* OMVs have the function of promoting the production of G-CSF, GM-CSF, IL-6, and IL-8 in human oral keratinocytes (HOK-16B cell lines). These bioactive molecules may act as potent immune stimulators, leading to periodontal inflammation (74). Apart from cells in the epithelial layer, BEVs can also act on the cells within the deep connective tissue, thereby triggering inflammatory responses. For example, the OMVs of *T. forsythia* stimulate the release of IL-6, IL-8, and monocyte chemoattractant protein-1 (MCP-1) from human periodontal ligament fibroblasts (hPDLCs) in a dose-dependent manner, and these pro-inflammatory factors levels are much higher than those induced by *T. Forsythia* itself (75).

As key effector cells in host defense, macrophages play a crucial role in combating microbial invasion, primarily through phagocytosis (76). Upon external stimulation, macrophages in the M0 basal state can polarize into distinct subtypes (77). M1 macrophages produce pro-inflammatory cytokines and promote osteoclasts formation, exacerbating periodontal inflammation, while M2 macrophages release anti-inflammatory cytokines to counteract the disease (78). OMVs from *F. nucleatum* promote macrophage polarization towards the pro-inflammatory M1 phenotype, further exacerbating the inflammatory environment and enhancing the toxicity of *F. nucleatum* OMVs toward mouse gingival fibroblasts (MGFs) (79). Host monocytes, as well as M (naïve) and M (IFNγ)-polarized macrophages, bind and phagocytose periodontal pathogen-derived OMVs, including those from *P. gingivalis*, *T. forsythia*, and *T. denticola* (80). This process activates NF-κB and inflammasome complexes, increasing the expression of inflammatory mediators such as tumor necrosis factor (TNF)-α, IL-8, and IL-1β. In another study, the stimulatory impacts of *P. gingivalis* EVs on macrophages were further verified. It was found that *P. gingivalis* OMVs could prompt macrophages to generate significantly higher levels of TNF-α, IL-12p70, IL-6, and IL-10, as well as interferon β (IFNβ) and nitric oxide (NO), compared with P. gingivalis alone. Simultaneously, OMV-stimulated macrophages were effectively able to activate caspase-1, resulting in the production of substantial amounts of IL-1β and IL-18. These macrophages released lactate dehydrogenase and exhibited 7-Aminoactinomycin D (7-AAD) positivity, which are clear indications of pyroptotic cell death (81). OMVs from *T. forsythia* activated the human monocytic cell line U937, producing inflammatory mediators with more pronounced inflammatory responses than those triggered by *T. forsythia* cells alone (75). *F. alocis* OMVs can also significantly increase the expression of cytokines such as C-C motif chemokine (CCL)1, CCL2, macrophage inflammatory protein-1 (MIP-1), CCL5, IL-1β, IL-6, IL-8, and TNF-α in human monocyte-derived THP-1 cells (74).

#### 4.2.2 Promotion of immune evasion

In addition to their pro-inflammatory effects, certain periodontal pathogens also secrete EVs that inhibit inflammation, allowing them to evade host immune defenses. It has been shown that *P. gingivalis* OMVs promote the loss of the LPS receptor CD14 on macrophages, with gingipains playing a key role in this process (82). This procedure impairs the macrophage response to LPS from *Escherichia coli* (*E. coli*) and reduces inflammation. Waller (83) et al. demonstrated that *P. gingivalis* EVs selectively promoting TNF tolerance via a TLR4- and mTOR-dependent mechanism, blocking host immune responses to the parent cells and facilitating local immune evasion. Moreover, *P. gingivalis* OMVs can selectively trap and activate neutrophils, initiating degranulation without being destroyed (84). They also degrade antimicrobial granule components, such as antimicrobial peptide LL-37 and myeloperoxidase, thereby protecting bacteria from being killed (84). OMVs from *A.actinomycetemcomitans* can act as decoys for immune cells, activating the complement system in an LPS-dependent manner and consuming complement components to protect susceptible bacteria in host serum (85). Such function has also been demonstrated for OMVs released by *P. gingivalis* (86). Choi (60) et al. found that OMVs secreted by major periodontal pathogens (*A. actinomycetemcomitans*, *P. gingivalis*, *T. denticola*) can transfer msRNAs to T cells, suppressing the expression of certain inflammation-related cytokines.

### 4.3 Periodontal tissue destruction

**Figure 4:**
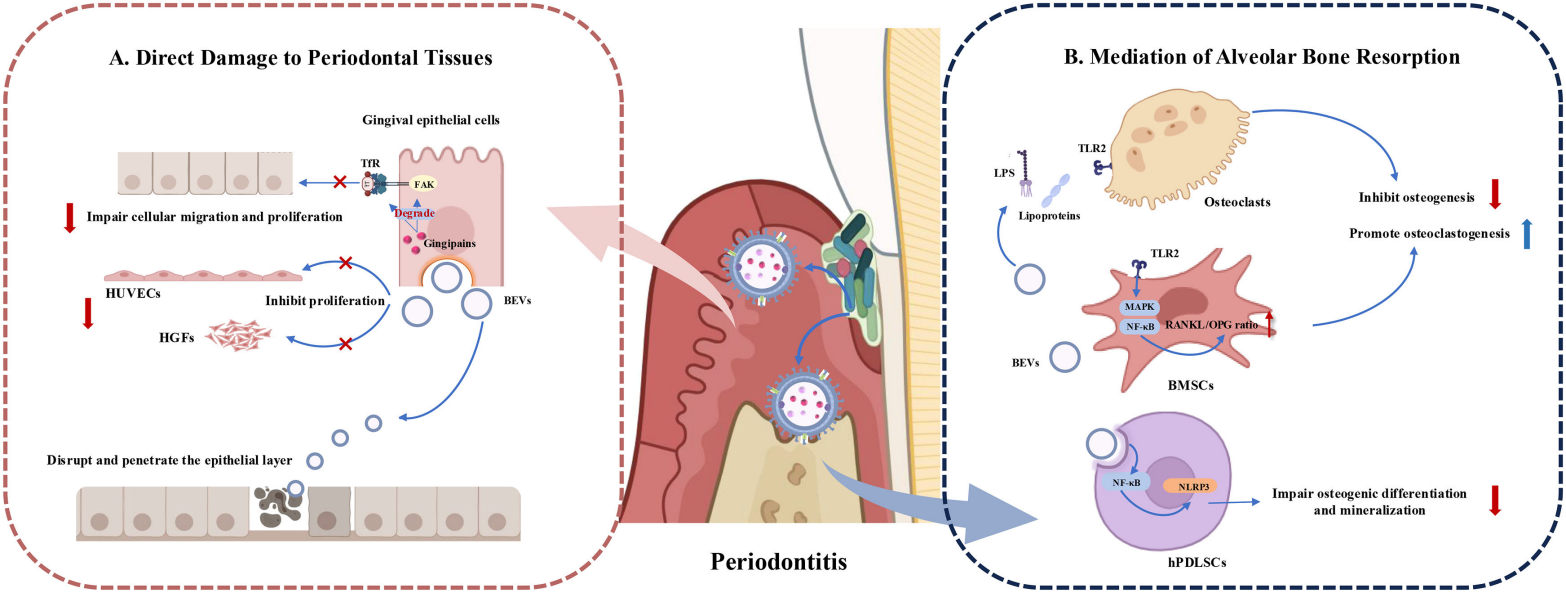
Roles of Periodontopathogen-Derived BEVs in Periodontal Tissue Destruction**. (A)** BEVs cause periodontal tissue destruction by impairing bioactivity of periodontal tissue cells such as gingival epithelial cells, human gingival fibroblasts (HGFs) and human umbilical vein endothelial cells (HUVECs). BEVs can also disrupt and enter the epithelial layer to play toxic roles. **(B)** BEVs induce bone destruction by releasing virulence factors and influencing the function of bone marrow stromal cells (BMSCs) and osteoclasts, to promote osteoclastogenesis and inhibit osteogenesis. BEVs can also be endocytosed by human periodontal ligament stem cells (hPDLSCs) and activate the NLRP3 inflammasome via the NF-κB (p65) signaling pathway. influencing osteogenic differentiation and mineralization.

In addition to their immunomodulatory effects, periodontal pathogen-derived BEVs can also exert the ability to destroy periodontal tissues through various mechanisms. The process of periodontal destruction by BEVs includes direct damage and invasion of the epithelial barrier, inhibition of angiogenesis, and the creation of an immunological microenvironment that induces bone resorption (**Figure 4**).

#### 4.3.1 Direct damage to periodontal tissues

EVs produced by Gram-negative bacteria can directly fuse with target cells or be internalized via lipid rafts, micropinocytosis, and clathrin-dependent endocytosis (87). Upon entering host cells, BEVs exhibit multiple virulence factors, exerting toxic effects on periodontal tissue cells, including gingival epithelial cells, vascular endothelial cells, and gingival fibroblasts (31).

Gingival epithelial cell layer is the first barrier to prevent periodontal pathogens from invading deep periodontal tissues (88). The close connection between the gingival epithelial cells ensures the integrity of the gingival epithelial barrier. OMVs from *P. gingivalis* rapidly enter host epithelial cells, such as HeLa cells and immortalized human gingival epithelial cells, via endocytosis (50). Gingipains associated with OMVs degrade functional molecules like transferrin receptor (TfR) and integrin-related signaling molecules (such as paxillin and focal adhesion kinase (FAK)), inhibiting the migration and proliferation of gingival epithelial cells and leading to cellular impairment (89). *T. denticola* OMVs can also disrupt and penetrate the epithelial layer (90). OMVs from *A. actinomycetemcomitans* fuse with lipid rafts on the plasma membranes of HeLa cells and HGFs, releasing cytolethal distending toxin (CDT), which remains biologically active in the nucleus and exerts cytotoxic effects (42).

Additionally, *P. gingivalis* OMVs dose-dependently inhibit the proliferation of human gingival fibroblasts (HGFs) and human umbilical vein endothelial cells (HUVECs), reducing their capillary formation ability and promoting periodontal disease progression, thus negatively affecting periodontal tissue regeneration (91).

#### 4.3.2 Mediation of alveolar bone resorption and destruction

BEVs from periodontal pathogens can influence osteoblasts and osteoclasts through various mechanisms, leading to an imbalance in periodontal bone homeostasis and exacerbating alveolar bone resorption. Indirectly triggering bone destruction by inducing inflammatory responses is a common mechanism. Studies have shown that EVs from *P. gingivalis*, *T. forsythia*, *Streptococcus oralis* (*S. oralis*), and *F. alocis* preferentially activate TLR2 to induce osteoclastogenesis, with aberrant osteoclast activation, consequently cause bone metabolism imbalance and eventual alveolar bone loss (92, 93). Likewise, BEVs produced from Gram-negative periodontal pathogens act on TLR2 primarily through lipoproteins and/or LPS (93). Additionally, *F. alocis* effectively activates the MAPK and NF-κB signaling pathways downstream of TLR2 and increases the RANKL/OPG ratio in bone-derived mesenchymal stromal cells (BMSCs), thus promote osteoclast differentiation, inhibit osteoblast differentiation, and lead to enhanced bone resorption (94). OMVs from *P. gingivalis* can also be internalized by human periodontal ligament cells (hPDLCs), inducing apoptosis and promoting alveolar bone resorption, a process regulated by microRNA-sized small RNAs (msRNA) such as sRNA45033 in *P. gingivalis* OMVs, which modulate DNA methylation (61). *F. nucleatum* OMVs enter human periodontal ligament stem cells (hPDLSCs) through endocytosis and activate the NLRP3 inflammasome via the NF-κB (p65) signaling pathway. This activation stimulates a series of cascade reactions, which subsequently results in osteogenic differentiation and mineralization impairment of hPDLSCs. Rat periodontitis models also proved *F. nucleatum* OMVs are important stimulators for alveolar bone loss (72).

## 5 Applications of BEVs in periodontitis

BEVs embody the dual nature of a double-edged sword. On the one hand, they contribute to the pathogenesis and are implicated in the onset and progression of diseases. On the other hand, they play a role in therapeutics, paving the way for innovative approaches to the diagnosis and treatment of related conditions.

### 5.1 BEVs for diagnosing periodontitis

Oral biofluids, such as saliva and gingival crevicular fluid (GCF), are rich of biomolecules from both host cells and resident microorganisms, and are commonly used to identify diagnostic markers for periodontitis (95). EVs found in these fluids are emerging as potential biomarkers for periodontal diseases (96–102). While most current research focuses on human-derived components, much less is known about BEVs. Han (102) et al. utilized LPS to label bacterial OMVs in saliva and found a significant increase in the amount of LPS+ OMVs in the saliva of patients with periodontitis compared to healthy individuals. Quantitative PCR analysis of genomic DNA from saliva small EVs (sEVs) indicated a marked increase in four periodontal pathogens (*T. denticola, E. corrodens, P. gingivalis, and F. nucleatum*) in the periodontitis group, with *P. gingivalis* and *T. denticola* being the most sensitive. These findings suggest that LPS+ OMVs, along with OMVs from *P. gingivalis* and *T. denticola*, could serve as potential diagnostic biomarkers for periodontitis. However, this study had a small sample size, and pure OMVs from specific periodontal pathogens were not isolated, warranting further investigation.

Other studies have explored the expression of virulence factors in OMVs as a diagnostic tool. For example, monoclonal antibodies have been developed to recognize the conserved *P. gingivalis* virulence factor RgpA-Kgp complex, forming the basis for a saliva-based diagnostic kit to detect *P. gingivalis* and its OMVs (103). However, the challenge remains in distinguishing BEVs from human-derived EVs in saliva or GCF due to the lack of specific markers (104). Therefore, separating BEVs for clinical diagnosis remains complex and difficult.

### 5.2 BEVs in vaccine development for periodontal diseases

BEVs are highly stable under various temperature and treatment conditions, and do not possess the ability to self-replicate, thus providing strong biosafety profiles (105). Due to their nanoscale size, BEVs are preferentially taken up by dendritic cells (DCs) (106). They also contain numerous immunogenic surface- and membrane-associated components of their parent bacterium, which can induce host immune responses (107, 108). Consequently, BEVs hold great promise as immunogenic biological agents that activate the immune system to combat bacterial infections. Moreover, BEVs can be bioengineered to express target antigens with reduced toxicity (109), making them promising candidates for vaccine development (110, 111).

For example, OMVs derived from *P. gingivalis* maintain the immunodominant epitopes of the bacterium, while animal studies by Nakao et al. (112) have demonstrated that intranasal administration of *P. gingivalis* OMVs in mice induced the dose-dependent production of salivary IgA, as well as serum IgG and IgA. Previous studies have estabilished that Poly(I:C), a TLR3 agonist, significantly increased antibody production and enhanced the clearance of *P. gingivalis* (112, 113). Moreover, the study confirmed the safety of low-dose intranasal immunization for adjacent organs and the central nervous system (113). The strong immunogenicity of *P. gingivalis* OMVs mainly originates from LPS and A-LPS-modified proteins, such as gingipains in the OMVs, while immune reactivity was significantly reduced after serum absorption of LPS (114).

Despite the promising progress, BEVs are still far from being used in clinical settings for the prevention of periodontal diseases. This is primarily due to the high toxicity of BEVs, as well as difficulties in isolating and characterizing them. Therefore, standard methods are urgently needed to isolate and characterize BEVs.

### 5.3 BEVs for drug delivery

Traditional antibiotic administration delivers drugs systemically via the bloodstream, allowing them to target pathogenic bacteria located in different parts of the body. However, this approach often lacks specificity, requiring higher doses and increased frequency to reach therapeutic concentrations at the infection site, which can lead to side effects and accelerate the development of antibiotic resistance (115). In recent years, the use of nanoparticle-based drug delivery systems has been extensively studied. These systems can enhance drug solubility, modulate drug release, target specific sites, and simultaneously deliver multiple therapeutic agents (116). Compared to synthetic nanoparticle carriers, naturally derived BEVs offer several advantages, including small particle size, stable cargo-carrying capacity, and high biocompatibility (117). BEVs possess immunogenic antigens on their surface, which can elicit robust immune responses against invading pathogens (107).

Additionally, BEVs are involved in signal transduction, facilitating inter-bacterial communication and exchange, and can easily fuse with bacterial membranes (118), delivering bioactive molecules to their parent bacteria and surrounding microbes. Through genetic engineering of parent bacteria, BEVs can be modified with targeting ligands, enhancing drug accumulation at desired sites (119). BEVs themselves can also be surface-modified to improve cellular or site-specific targeting (120). Accordingly, BEVs have been explored as drug delivery vehicles to enhance bacterial uptake of loaded antibiotics. Previous evidence suggests that BEVs are more potent in delivering autolysins and peptidoglycan hydrolases, resulting in higher bacterial killing efficiency compared to gentamicin (121). Another study developed a novel antibiotic delivery system using OMVs isolated from *E. coli* as a shell and rifampicin-loaded mesoporous silica nanoparticles (MSNs) as the core. In contrast to conventional antibiotics, BEVs could enhance antibiotic uptake and achieve superior antibacterial effects (122).

Currently, the mechanisms of BEV-mediated delivery are not fully understood, and there is a need to improve purification processes and production yields, as well as to standardize the techniques and analyses (123). While the application of BEV-based drug delivery systems in clinical practice remains distant, BEVs hold great promise as novel antibiotic delivery vehicles or potential antimicrobial agents to effectively kill or inhibit periodontal pathogens.

### 5.4 BEVs inhibitors for inflammation control

Periodontopathic bacteria-derived EVs contain various toxic factors that contribute significantly to periodontal tissue destruction, as discussed previously. Inhibiting the release of these EVs from periodontal pathogens could represent a therapeutic approach to managing periodontitis. Peptidylarginine deiminases (PADs), a group of calcium-activated enzymes, serve as toxic components in *P. gingivalis* EVs. PADs convert arginine residues into citrulline residues, which lead to the citrullination of host proteins such as fibrinogen and α-enolase. This modification is crucial for various physiological processes, including the biogenesis of OMVs and the initiation of pathological inflammation (124). The use of PAD inhibitors, such as GSK 199, BB-Cl-amidine, Cl-amidine, and AMF30a, has been shown to effectively reduce BEVs production (125, 126). Additionally, cannabidiol (CBD) has been reported to inhibit the release of BEVs from Gram-negative bacteria, potentially reducing antibiotic resistance (127).

Given BEVs’ ability to mediate immune-inflammatory responses and promote periodontitis progression and tissue damage, finding agents to reduce the release of pro-inflammatory factors induced by BEVs opens new therapeutic avenues. Hop bract polyphenol (HBP), for example, has been shown to inhibit the expression of cyclooxygenase (COX)-2, IL-6, IL-8, and matrix metalloproteinases (MMP)-1 and -3 in human gingival epithelial (HGE) cells challenged with *P. gingivalis* EVs in a dose-dependent manner. This makes HBP a promising inhibitor of the cell inflammatory response induced by *P. gingivalis* EVs. Key active components of HBP, such as 2-[(2-methylpropanoyl)-phloroglucinol]1-O-β-D-glucopyranoside (MPPG) and kaempferol 3-O-β-glucopyranoside (astragalin), have been identified as effective in mediating these anti-inflammatory effects (128). Curcumin also shows notable efficacy, significantly inhibiting *P. gingivalis* OMV-stimulated gene expression and protein production of IL-6, IL-1β, and TNF-α in HGE cells, in a dose-dependent manner. Additionally, curcumin attenuates the cytotoxic effects of OMVs on cell migration and reduces OMV adhesion, cell entry, and apoptosis, also in a dose-dependent manner (129).

Probiotic therapy aimed at neutralizing the toxic effects of periodontal pathogen-derived EVs may represent an emerging area of research. Microbial dysbiosis is critical in the progression of periodontitis, typically involving a reduction in probiotic bacteria and/or an increase in periodontal pathogens (130). *Lactobacillus reuteri* (*L. reuteri)* is one of the most extensively studied probiotics and shows potential as an adjunctive treatment for chronic periodontitis (131). *L. reuteri* has been found to downregulate key virulence factors of periodontal pathogens, interfere with interspecies communication among pathogens, inhibit pathogenic adhesion and invasion, and reduce virulence (132). Probiotic-derived EVs also possess anti-infective properties (133). Notwithstanding current evidence on the efficacy of probiotic-derived EVs in periodontal inflammation control is limited, this approach does indicate a promising direction for future research.

## 6 Conclusion and outlook

Extracellular vesicles (EVs) secreted by key periodontal pathogens play a pivotal role in the interactions between bcteria and between bacteria and the host, thereby influencing periodontal homeostasis and contributing to the progression of periodontitis. This review summarizes the production, composition, and biological characteristics of BEVs from periodontal pathogens and discusses recent advances regarding their involvement in the pathogenesis, diagnosis, treatment, and prevention of periodontitis.

BEVs originate from parent bacterial cells, which carry and transmit a variety of virulence factors. On one hand, BEVs can induce local immune-inflammatory responses, while on the other, they promote immune evasion by suppressing immune surveillance and facilitating the proliferation of parent bacteria. BEVs promote bacterial co-aggregation and play a role in biofilm pathogenicity. In periodontitis, BEVs can directly damage periodontal connective tissues and, through various mechanisms, indirectly mediate alveolar bone resorption and destruction, thus contributing to disease progression.

Given their widespread presence in gingival crevicular fluid and saliva, BEVs have been investigated as potential biomarkers for periodontitis. Moreover, their biological properties suggest they are promising candidates for vaccine development and drug delivery systems. However, current research on the components and pathogenic mechanisms of BEVs remains insufficient. Specific markers for distinguishing and isolating EVs from different sources are lacking, and standardized protocols for the production and purification of BEVs still need to be developed.

In the future, further research on BEVs is expected to deepen our understanding of their role in periodontal diseases. More efforts should be made to clarify the detailed composition and specific pathogenic mechanisms of BEVs. This knowledge could guide the development of targeted therapies that mitigate BEV-mediated inflammation and tissue destruction. Identifying specific molecular markers that are unique to periodontal pathogen-derived BEVs is crucial to enhance diagnostic precision and therapeutic targeting. Developing cost-effective and standardization of BEVs preparation processes will enhance the reliability and reproducibility of their applications as vaccine candidates and drug delivery materials.

Moreover, exploring new strategies to modulate the activity of BEVs may provide novel therapeutic approaches for periodontitis. For example, developing novel drugs that can block the harmful effects of BEVs or enhance their beneficial functions could hold promise. The immunomodulatory potential of probiotic-derived EVs represents an innovative area for managing chronic periodontitis. Future research should assess the anti-inflammatory effects and mechanism of action of probiotic-derived EVs to determine their potential as adjunct therapies in periodontitis treatment. Additionally, combining BEVs with other advanced technologies such as nanotechnology and immunotherapy, may lead to more effective treatment options.

As our knowledge of BEVs continues to expand, their potential applications in periodontal diagnosis, treatment, and prevention will become more prominent, ultimately contributing to better management of periodontal diseases and improve oral health.
